# A potential pathogenic role of interleukin-6 in a child with ANCA-negative pauci-immune crescentic glomerulonephritis: case report and literature review

**DOI:** 10.1186/s12882-021-02504-w

**Published:** 2021-08-23

**Authors:** Ling Hou, Lu Yin, Yubin Wu, Chengguang Zhao, Yue Du

**Affiliations:** Department of Pediatrics, Shengjing Hospital of China Medical University, No.36 Sanhao Street, Heping District, Shenyang, 110004 China

**Keywords:** Anti-neutrophil cytoplasmic antibody, Pauci-immune, Crescentic glomerulonephritis, Child

## Abstract

**Background:**

Crescentic glomerulonephritis is a disease characterized by severe glomerular injuries that is classified into five different pathological types. Patients with type V disease have pauci-immune crescentic glomerulonephritis (PICGN) that is negative for anti-neutrophil cytoplasmic autoantibodies (ANCAs). There are limited clinical data on the manifestations, treatment, and prognosis of type V crescentic glomerulonephritis, especially in children.

**Case presentation:**

A 13-year-old girl who had an intermittent fever for more than 10 months was admitted to our hospital. She had no gross hematuria, oliguria, edema, or hypertension, but further tests indicated a decreased glomerular filtration rate, hematuria, proteinuria, and an elevated level of IL-6. The antinuclear antibody spectrum test was positive at 1:1000, and the ANCA and anti-glomerular basement membrane antibody tests were negative. A renal biopsy confirmed the diagnosis of ANCA-negative PICGN. We administered methylprednisolone pulse therapy with intravenous cyclophosphamide and oral mycophenolate mofetil. At the 3-month follow-up, her urine protein level was significantly lower, and her serum creatinine level was in the normal range.

**Conclusions:**

Fever may be an extrarenal manifestation of ANCA-negative PICGN, and IL-6 may play a role in the pathogenesis of this disease. Early methylprednisolone pulse therapy with an immunosuppressant may reduce symptoms and improve prognosis.

## Background

Crescentic glomerulonephritis (GN) is not a specific disease but a histological manifestation of severe glomerular damage, which defines a group of diseases. GN is usually clinically associated with macroscopic or microscopic hematuria, erythrocyte casts, a variable degree of proteinuria, and loss of renal function upon onset. Nephritic syndrome and rapidly progressive GN are common features in all types of crescentic GN. This condition is characterized by a severe glomerular injury in which 50% or more of the glomeruli appear as extensive crescents. There are different precipitating factors and different types. However, in all cases, the glomerular capillary loops are damaged, the glomerular basement membrane (GBM) is ruptured, and this damage causes the proliferation of glomerular epithelial cells and infiltration of macrophages that form cellular crescents. In the later stage of disease, due to transdifferentiation of the epithelial cell phenotype and extracellular matrix formation, there is a gradual transformation into fibro cellular and fibrous crescents [1]. The renal outcome is often unfavorable, and more than 80% of patients progress to end-stage renal disease (ESRD) within 3 months from the onset of illness [2]. There are five types of crescentic glomerulonephritis: type I (anti-GBM disease) refers to the accumulation of antibodies against collagen in the GBM; type II refers to the deposition of immune complexes in the glomeruli; type III (pauci-immune) refers to small-vessel vasculitis due to anti-neutrophil cytoplasmic antibodies (ANCAs); type IV refers to different combinations of types I and III; and type V refers to ANCA-negative pauci-immune crescentic glomerulonephritis (PICGN) [3].

The incidence of ANCA-negative PICGN is about 14% in children with renal vasculitis, and only one-third of them have a crescentic histological pattern [4]. Published data regarding kidney involvement and prognosis are scarce. The present case report describes the successful treatment of a 13-year-old girl with ANCA-negative PICGN and reviewed previous case reports of this condition in children [5–11].

## Case presentation

In August 2020, a 13-year-old girl was admitted to our hospital due to prolonged and intermittent fever. She first developed a fever 10 months previously that lasted for 7 days. During that time, her body temperature was elevated twice per day, reached as high as 40 °C, and was accompanied by nausea, vomiting, abdominal pain, and chills, but no rash. Subsequently, she had a fever almost once per month, lasting for about 5 to 7 days and resolving after antipyretic treatment.

Previous examinations at the local hospital (December 2019) determined her white blood cell (WBC) count was 10.18 × 10^9^/L (75.1% neutrophils), eosinophil count was normal (0.13 × 10^9^/L, reference range: 0.04 ~ 0.49 × 10^9^/L), hemoglobin was 123 g/L, platelet count was 213 × 10^9^/L, and C-reactive protein (CRP) level was 13.22 mg/L (reference range: 0 ~ 8 mg/L). Then in March 2020, a routine blood test indicated a WBC count of 7.01 × 10^9^/L (63.6% neutrophils), hemoglobin of 107 g/L, platelet count of 227 × 10^9^/L, and CRP of 79.6 mg/L. There were no microbiological tests.

She came to our hospital at that time because her fever was frequent and the duration was prolonged. She also had a history of right upper limb fractures after two falls when she was 6 and 10 years old. There were no abnormalities in her personal or family history and no abnormal signs on admission (bodyweight: 66 kg, stable from disease onset; blood pressure: 126/81 mmHg; height: 170 cm). The blood pressure in her extremities was symmetrical, and she had no abnormal vascular murmur.

Laboratory results in August 2020 (Tables 1 and 2) indicated that the WBC count and neutrophils were in the normal ranges, and the hemoglobin was slightly low. Indicators of infection and inflammation, including procalcitonin (PCT), CRP, and erythrocyte sedimentation rate (ESR), were obviously increased. We also measured the levels of multiple cytokines. The level of interleukin (IL) -6 was significantly elevated, the level of IL-10 was slightly elevated, but the levels of IL-2, IL-4, IL-17, INF-γ, and TNF were normal. Tests for mycoplasma and herpes simplex virus-IgM antibodies were positive. Screening for tumor markers, such as neuron-specific enolase (NSE) and alfa-fetoprotein (AFP), indicated no abnormalities. Bone marrow aspiration results were negative. Pulmonary computerized tomography (CT) and whole abdominal enhanced CT results indicated no apparent abnormalities, while echocardiography and subclavian artery ultrasound were also normal. We administered anti-infective therapy with ceftriaxone and azithromycin after hospitalization. After 3 days, her body temperature returned to normal, and her CRP level decreased to 40.4 mg/L.

**Table 1:** Laboratory test results at admission

Variable	Result	Variable	Result	Variable	Result
WBC, 10^9^/L	8.62	PCT, ng/mL	**0.379**	IgM, g/L	1.25
Hb, g/L	**102**	CRP, mg/L	**114.00**	IgE, KU/L	**1693**
HCT, %	**30.9**	ESR, mm/h	**96**	IgG4, g/L	0.681
PLT, 10^9^/L	273	ASO, IU/mL	44.7	C3, g/L	1.22
TP, g/L	66.9	MP-IgM	**+**	C4, g/L	0.194
ALB, g/L	**32.6**	HSV-IgM	**+**	ANA	**1:1000**
ALT, U/L	9	HBV	–	dsDNA	–
AST, U/L	13	HCV	–	Anti-GBM Ab	–
LDH, U/L	249	HIV	–	PR3-ANCA	–
T-Bil, μmol/L	4.8	Ferritin, ng/mL	279.0	cANCA	–
T-Cho, mmol/L	4.96	NSE, ng/mL	40.840	MPO-ANCA	–
TG, mmol/L	0.69	AFP, ng/mL	< 0.605	pANCA	–
BUN, mmol/L	5.34	VMA, mg/day	4.75	PLA2R-IgG	–
SCr, μmol/L	**87.9**	PT, s	11.3	Urinalysis	
UA, μmol/L	323	APTT, s	37	pH	6.00
Na, mmol/L	141	Fib, g/L	**5.1**	s.g.	1.014
K, mmol/L	3.98	FDP, mg/L	**5.8**	RBC casts	**2**
Cl, mmol/L	108.5	D-dimer, μg/L	**691**	RBC/HPF	**34.7**
Ca, mmol/L	**2.04**	IgA, g/L	1.32	Dysmorphic RBC, %	**60**
Glu, mmol/L	4.25	IgG, g/L	13.90	WBC, /HP	15.62
				Urinary protein, g/day	**1.90**

Bold numbers indicate a level outside the reference range

*WBC* White blood cells, *Hb* Hemoglobin, *HCT* Hematocrit, *PLT* Platelets, *TP* Total protein, *ALB* Albumin, *ALT* Alanine transaminase, *AST* Aspartate transaminase, *LDH* Lactate dehydrogenase, *T-Bil* Total bilirubin, *T-Cho* Total cholesterol, *TG* Triglycerides, *BUN* Blood urea nitrogen, *SCr* Serum creatinine, *UA* Uric acid, *Glu* Glucose, *PCT* Procalcitonin, *CRP* C reactive protein, *ESR* Erythrocyte sedimentation rate, *ASO* Antistreptolysin O, *MP* Mycoplasma, *HSV* Herpes simplex virus, *HBV* Hepatitis B virus, *HCV* Hepatitis C virus, *HIV* Human immunodeficiency virus, *NSE* Neuron-specific enolase, *AFP* Alpha-fetoprotein, *VMA* Vanilla mandelic acid, *PT* Prothrombin time, *APTT* Activated partial thromboplastin time, *Fib* Fibrinogen, *FDP* Fibrinogen degradation products, *ANA* Antinuclear antibody, *dsDNA* Double-stranded DNA, *GBM* Glomerular basement membrane, *ANCA* Anti-neutrophil cytoplasmic antibody, *PLA2R* Anti-phospholipase A2 receptor antibodies. *s.g*. Specific gravity

**Table 2:** Cytokine levels at admission

Variable	Result	Reference range
IL-2, pg/mL	1.33	1.07 ~ 1.43
IL-4, pg/mL	1.13	1.13 ~ 1.71
IL-6, pg/mL	**40.04**	2.15 ~ 12.75
IL-10, pg/mL	**3.44**	1.48 ~ 2.16
IL-17, pg/mL	3.60	5.06 ~ 10.06
INF-γ, pg/mL	1.28	2.55 ~ 4.37
TNF, pg/mL	1.37	1.05 ~ 1.95

*IL* Interleukin, *INF* Interferon, *TNF* Tumor necrosis factor

At 2 days after admission, her serum creatinine was slightly increased at 87.9 μmol/L (reference range: 33 ~ 75 μmol/L), and 99mTc-DTPA renal dynamic imaging indicated the glomerular filtration rate (GFR) was 74.4 mL/min/1.73 m^2^ (reduced renal function). Urinalysis indicated hematuria and proteinuria, and analysis of urinary sediment indicated 2 erythrocyte casts per HPF and 34.7 RBCs per HPF, with a dysmorphic rate of 60%. She tested positive for ANA (1:1000), but negative for anti-double-stranded DNA, anti-GBM, ANCA (PR3-ANCA, cANCA, MPO-ANCA, pANCA), and anti-phospholipase A2 receptor antibodies (PLA2R). The complement C3 and C4 levels were in the normal range, although the serum IgE was increased. Kidney ultrasound showed that both kidneys were enlarged with increased renal cortex echoes and blurred cortical and medullary boundaries.

**Fig. 1:**
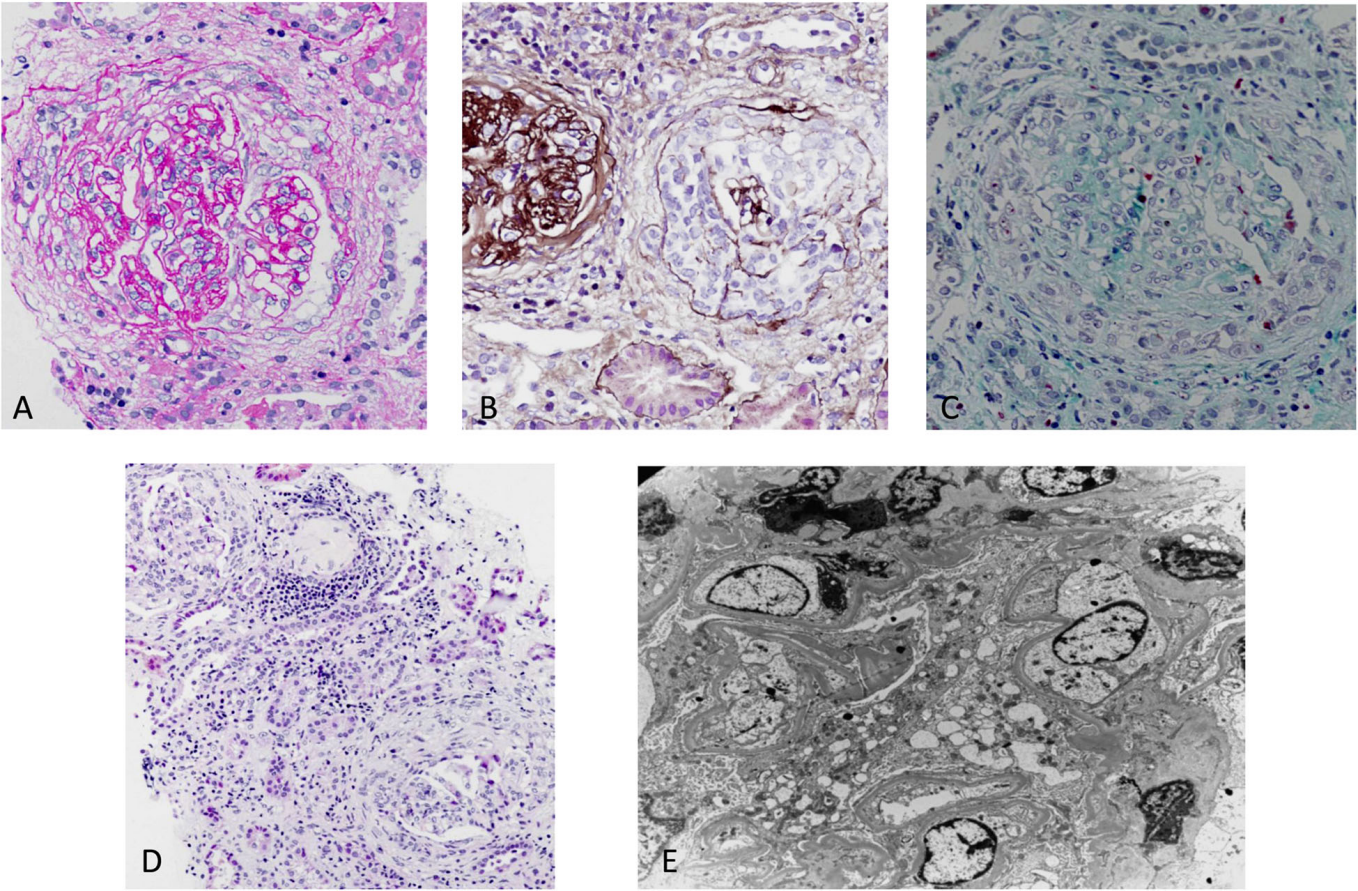
Representative histopathologic sections from a renal biopsy of 25 glomeruli. Light microscopy (**A**–**D**) shows cellular fibrous crescents, cellular crescents and tubular atrophy, interstitial fibrosis, and massive infiltration of lymphocytes and monocytes. Electron microscopy (**E**) shows a slight increase of mesangial matrix and a small amount of electron density scattered in the mesangial area. **A**, Periodic acid Schiff (PAS) staining (400×). **B**, Periodic-acid silver methenamine (PASM) staining (400×). **C**, Masson staining (400×). **D**, hematoxylin-eosin staining (200×). **E**, electron microscopy (4000×)

Therefore we performed a kidney biopsy and examined 25 glomeruli using microscopy (Fig. 1). Seven glomeruli had global sclerosis, 1 had cellular crescents, 11 had fibro cellular crescents, and 4 had fibrous crescents. The remaining glomerular aberrations were mild, with open capillary loops and no significant alterations in the mesangial area. However, the visceral and parietal epithelial cells were swollen, and Bowman’s capsule thickened and stratified. The renal tubular epithelial cells had granular degeneration, multifocal tubular atrophy (45%), the absence of tubular structures with interstitial fibrosis, and infiltration of lymphocytes and monocytes. There were no apparent changes in the arterioles. Immunofluorescence microscopy indicated 6 glomeruli had granular deposition of IgG (+) in the mesangial area and capillary wall, but the other results were negative (IgA−, IgM−, C1q−, Fib−, and C3±). Electron microscopy indicated one glomerulus had a slightly increased segmental glomerular mesangial matrix, a small number of electron-dense regions scattered in the mesangial area, swollen podocytes and parietal epithelial cells, and a collapsed capillary basement membrane. The thickness of the GBM was normal, and there was no evidence of delamination, tearing, or arachnoid changes. There were also no thickened inner lighter layers of the GBM. The mitochondria of the renal tubular epithelial cells were swollen, and the endoplasmic reticulum was expanded. The basement membranes of some tubules were thickened, and collagen fibers and a small number of monocytes and lymphocytes were present in the interstitium. There were no apparent changes in the arterioles.

Our results indicated the patient had ANCA-negative PICGN. We administered 2 courses of intravenous methylprednisolone pulse therapy (500 mg/day for 3 days each), and then oral prednisone (60 mg/day) with gradual tapering. We simultaneously administered intravenous cyclophosphamide (500 mg/day for 2 days each month) and oral mycophenolate mofetil (MMF, 0.75 in the morning, 0.5 in the evening). At the 3-month follow-up (November 2020), the child’s 24-h urine protein level decreased but remained slightly elevated at 0.59 g/day (previously 1.90 g/day), her serum creatinine decreased to the normal range (42.6 μmol/L; previously 87.9 μmol/L), and her serum albumin increased to the normal range (40.2 g/L; previously 32.6 g/L).

## Discussion

This case report describes a 13-year-old girl who had a long history of intermittent fever. Due to the prolonged duration of her condition, she frequently went to the hospital but received no systematic examination or diagnosis. We admitted her 10 months after she first developed a fever and initially considered whether specific pathogens caused the fever and complicated illnesses. Although the tests for herpes simplex virus and the mycoplasma pneumonia IgM antibodies were positive, her PCT and CRP levels were significantly increased. Thus, we first considered bacterial infection and administered azithromycin with cephalosporin. Her body temperature dropped to a normal level within 3 days. However, we could not confirm a specific pathogen because of her history of repeated fever over many months. Thus, we also tested for possible tumors using bone marrow aspiration, measurement of tumor markers, and various imaging examinations, but none of the results were positive. Examination of immunological indicators of autoimmune diseases indicated that only the ANA titer was significantly increased (1:1000). This suggested possible autoimmune involvement, but the family rejected genetic testing for economic reasons.

Before treatment, the girl had no urinary system symptoms, such as gross hematuria, oliguria, edema, or hypertension. However, after admission, a systemic examination indicated hematuria and proteinuria and a mild decrease in GFR. Urinalysis is a routine test for patients with fever, urine sediment, proteinuria, and microscopic hematuria, especially when autoimmune tests are negative. She had no progressive nephritic symptoms, such as progressive oliguria or hypertension, and her urine output and blood pressure were normal. A renal biopsy and analysis of 25 glomeruli indicated that 7 glomeruli had global sclerosis, 4 had fibrous crescents, 11 had fibro cellular crescents, and 1 had cellular crescents. About 45% of the renal tubules were atrophied with interstitial fibrosis and inflammatory cell infiltration. These results confirmed the presence of a severe kidney injury. A renal biopsy is essential for guiding therapy and predicting prognosis in patients with PICGN, and an early kidney biopsy provides a faster diagnosis and earlier initiation of treatment. Timely treatment of patients with active disease significantly improves short-term recovery and prognosis and is associated with disease remission. Thus, patients may recover quickly after a kidney biopsy, and a delayed diagnosis reduces the chances of successful treatment.

Because the patient had fibro-cellular and cellular crescents in 12 of 25 glomeruli and an increased level of ANA, we administrated methylprednisolone pulse therapy with cyclophosphamide and MMF. Subsequent urine protein and renal function measurements indicated significant improvements during the follow-up and no noticeable adverse reactions. The presence of crescents in the kidney biopsy suggested that there may have been repeated crescent formation. Thus, we concluded that her prolonged and repeated fever was most likely an extrarenal symptom of ANCA-negative PICGN, consistent with adult reports of ANCA-negative PICGN [12]. Previous research reported the renal survival was poorer in patients with ANCA-negative than ANCA-positive disease [12], possibly due to delayed diagnosis and less extrarenal involvement in ANCA-positive patients.

There is a low incidence of ANCA-negative PICGN in children. Shimizu et al. reported a 6-year-old girl with hematuria, proteinuria, skin rash, and arthralgia in the lower extremities [5]. She quickly progressed to renal insufficiency and required renal replacement therapy. They reported that her IL-6 was level significantly increased during the acute phase (28.4 pg/mL; their reference range: 0–4 pg/mL) but returned to the normal range after treatment. Similarly, our patient had a significantly increased level of IL-6 (40.04 pg/mL; our reference range: 2.15–12.75 pg/mL) with repeated fever, but it also returned to the normal range after treatment with cyclophosphamide and MMF. A previous report described a 76-year-old adult patient with multiple metastases from pulmonary adenosquamous carcinoma combined with ANCA-negative PICGN and an IL-6 level of 425 pg/mL (their reference range: 0–2.41 pg/mL) [13]. Another study described a 31-year-old male with seronegative pauci-immune necrotizing GN with an elevated serum IL-6 level (15.7 pg/mL; their reference range: 0–2.1 pg/mL) [14]. Our results and the results of these three previous studies suggest a possible role of the inflammatory factor IL-6 in the pathogenesis of ANCA-negative PICGN.

**Fig. 2:**
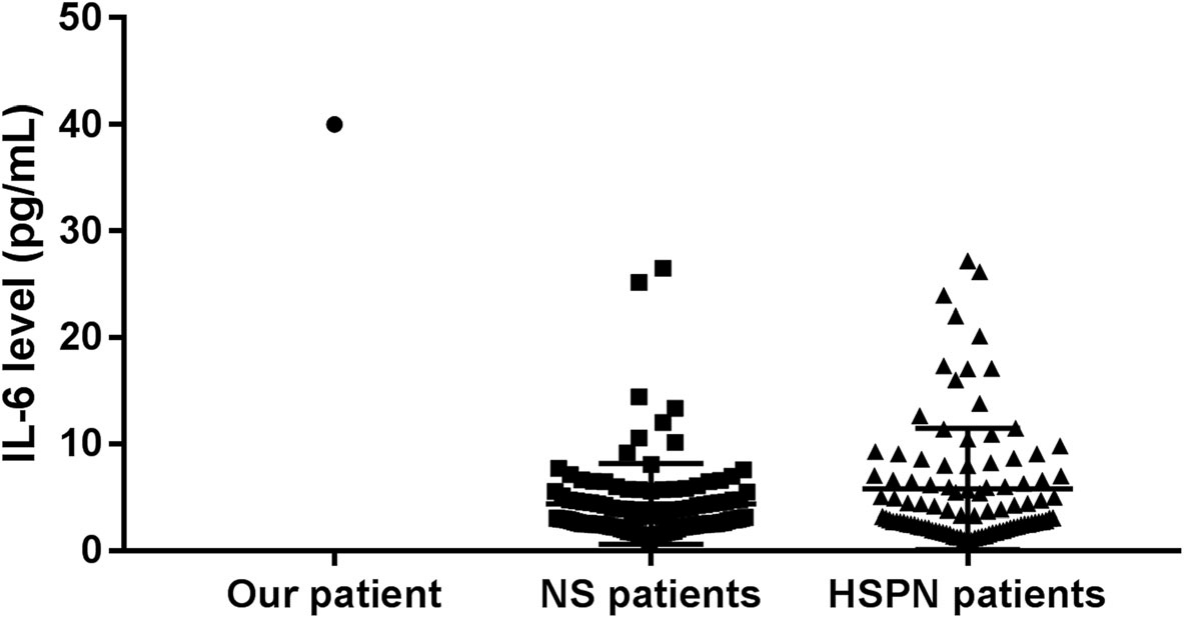
IL-6 levels in our patient, patients with nephrotic syndrome (*n* = 114), and patients with Henoch-Schönlein purpura nephritis (*n* = 100)

We also measured cytokines in children admitted to our center with diagnoses of nephrotic syndrome (NS; 114 patients) or Henoch–Schönlein purpura nephritis (HSPN; 100 patients). The levels of IL-2, IL-4, IL-10, and TNF were elevated in those with NS, and IL-17 was elevated in those with HSPN, both during the acute phase of disease. However, there was no elevation of IL-6 in patients with NS (4.37 ± 0.354 pg/mL, *n* = 114) or HSPN (5.79 ± 0.568 pg/mL, *n* = 100; Fig. 2).

IL-6 is a pleiotropic cytokine that plays a key role in regulating immune responses, inflammation, and hematopoiesis. An elevated IL-6 level also occurs in various immune diseases, such as rheumatoid arthritis and Castleman disease, and nephropathies in multiple myeloma [15]. Diabetic patients with diabetic nephropathy have higher serum IL-6 levels than those without diabetic nephropathy [16], and IL-6 participates in the pathogenesis and progression of diabetic nephropathy [17]. Lupus nephritis patients have significantly elevated levels of IL-6, and IL-6 can be used as a sensitive biomarker for disease activity and a predictor of remission in these patients [18]. IL-6 functions via classical signaling and trans-signaling, and trans-signaling plays a central role in crescentic GN in a nephrotoxic serum-induced murine model of nephritis [14]. In this animal model, the serum IL-6 level was significantly elevated, inhibition of the IL-6 pathway led to milder disease, and activation of this pathway significantly aggravated disease. In patients with ANCA-positive PICGN, IL-6 plays an essential role in neutrophil activation, mainly due to its induction of helper T lymphocytes that can produce IL-17 [19]. However, the IL-17 level was normal in our case and in the previously described adults with pulmonary adenosquamous carcinoma, indicating that IL-6 may not act by inducing IL-17 production in ANCA-negative PICGN. However, our results are from a single patient and do not prove a causal relationship between IL-6 and disease activity. The role and mechanism of IL-6 in ANCA-negative PICGN, therefore, needs further study.

## Conclusion

We described a 13-year-old girl who was diagnosed with ANCA-negative PICGN following a long-term fever. An elevated level of IL-6 may possibly contribute to the pathogenesis of this disease. Early combined treatment with methylprednisolone pulse therapy and an immunosuppressive agent may significantly alleviate the proteinuria and improve the renal function in these patients.

## Data Availability

The datasets used during the current study are available from the corresponding author on reasonable request.

## Abbreviations

ANCA: Anti-neutrophil cytoplasmic autoantibodies
PICGN: Pauci-immune crescentic glomerulonephritis
RPGN: Rapidly progressive glomerulonephritis
GBM: Glomerular basement membrane
WBC: White blood cell
CRP: C-reactive protein
PCT: Procalcitonin
ESR: Erythrocyte sedimentation rate
IL: Level of interleukin
NSE: Neuron-specific enolase
AFP: Alfa-fetoprotein
CT: Computerized tomography
GFR: Glomerular filtration rate
